# Microsatellite Instability‐High Somatic‐Type Malignancy Arising From a Primary Retroperitoneal Germ Cell Tumor: A Case Report

**DOI:** 10.1002/iju5.70243

**Published:** 2026-08-18

**Authors:** Gaku Hayashi, Tomokazu Kimura, Satoshi Inoue, Kazuna Matsuo, Tomoyasu Sano, Kenji Zennami, Shohei Ishida, Akira Satou, Shusuke Akamatsu

**Affiliations:** ^1^ Department of Urology Nagoya University Graduate School of Medicine Nagoya City Japan; ^2^ Department of Pathology and Laboratory Medicine Nagoya University Hospital Nagoya City Japan

**Keywords:** DNA mismatch repair, germ cell tumor, microsatellite instability, Pembrolizumab

## Abstract

**Introduction:**

Immune checkpoint inhibitors have shown only limited clinical efficacy in germ cell tumors (GCTs), underscoring the need for predictive biomarkers such as microsatellite instability‐high (MSI‐high). We report a rare case of mixed‐type GCTs treated with chemotherapy, in which the somatic‐type malignancy (STM) component was found to be MSI‐high.

**Case Presentation:**

A 55‐year‐old man presented with a retroperitoneal mass, and an open biopsy revealed a mixed GCT. He was diagnosed with primary retroperitoneal GCT and treated with four cycles of bleomycin, etoposide, and cisplatin chemotherapy. Fourteen months later, retroperitoneal lymph node enlargement was observed, and retroperitoneal lymph node dissection revealed an STM. Six months later, local recurrence had occurred. MSI testing of the resected recurrent tumor demonstrated an MSI‐high status.

**Conclusion:**

This case illustrates that careful selection of tissue specimens and appropriate timing of MSI testing are essential to identify potentially actionable cases in patients with GCTs.

## Key Clinical Message

MSI testing should be considered in refractory germ cell tumors using appropriate tissue specimens obtained from progressive or recurrent lesions after standard treatment.

## 1 Introduction

For refractory or relapsed germ cell tumors (GCTs) failing conventional chemotherapy, few effective alternatives exist [1]. Although immune checkpoint inhibitors (ICI)—particularly pembrolizumab [2, 3, 4]—have been trialed in patients with GCTs, clinical benefit remains minimal. GCTs generally show significantly lower response rates to PD‐1 inhibitors than other malignancies [5]. While predictive biomarkers like microsatellite instability‐high (MSI‐high) are crucial for anti–PD‐1 therapy, such GCTs remain extremely rare [6].

Herein, we report a case of mixed GCT with somatic‐type malignancy (STM) that developed after chemotherapy and was subsequently found to be MSI‐high.

## 2 Case Presentation

A 55‐year‐old man with no significant medical history presented to an emergency department with left lower abdominal pain in August 2021. Computed tomography (CT) revealed a retroperitoneal mass, and an open biopsy demonstrated a mixed germ cell tumor comprising seminoma, teratoma, and yolk sac tumor components.

**FIGURE 1:**
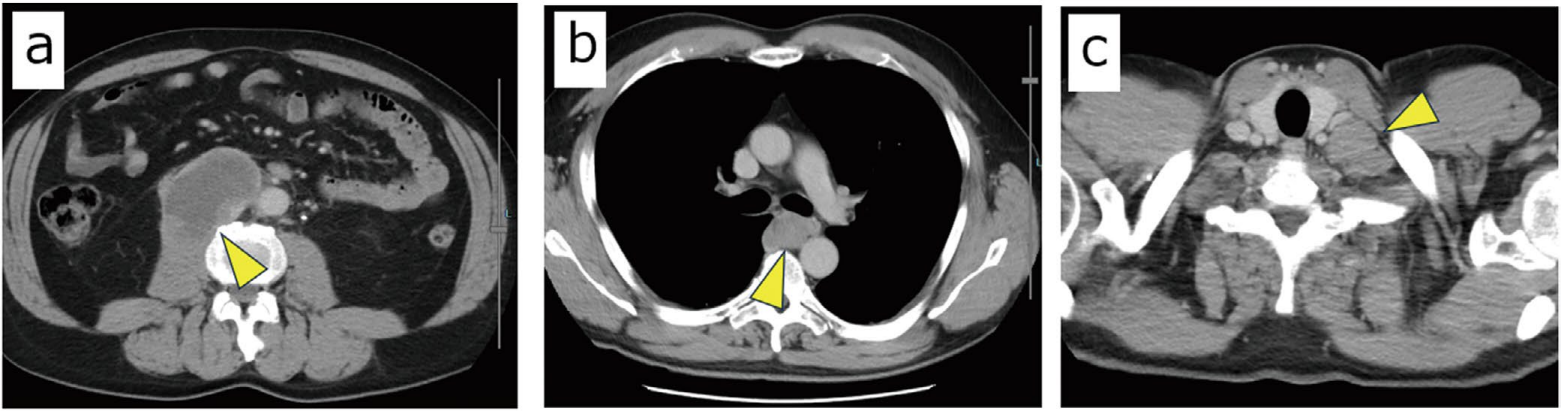
Contrast‐enhanced tomography (CT) revealed metastases to the left supraclavicular (c), mediastinal (b), and para‐aortic lymph nodes (a), accompanied by right ureteral obstruction. No other organ metastases were detected.

Testicular ultrasonography and magnetic resonance imaging demonstrated no abnormalities. CT revealed metastases to the left supraclavicular, mediastinal, and para‐aortic lymph nodes, accompanied by right ureteral obstruction. No other organ metastases were detected (Figure 1a–c). Laboratory testing showed a human chorionic gonadotropin (hCG) level of 8.7 IU/L, lactate dehydrogenase (LDH) 977 IU/L, alpha‐fetoprotein (AFP) 174.2 ng/mL, and a serum creatinine level of 1.29 mg/dL (eGFR, 45 mL/min/1.73 m^2^). A right ureteral stent was placed 2 days before the initiation of chemotherapy.

**FIGURE 2:**
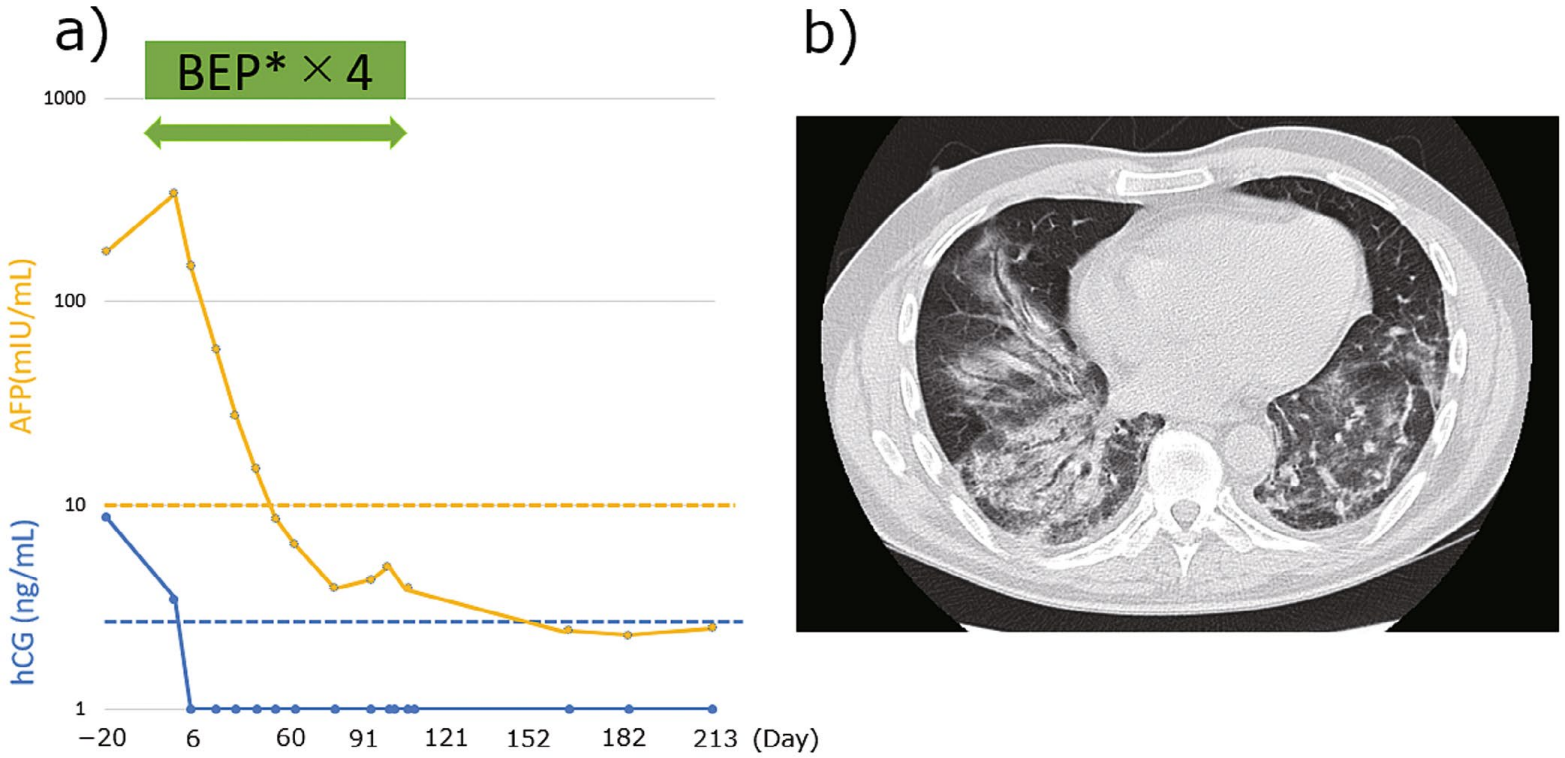
(a) Time course of AFP and hCG levels in this patient. The BEP chemotherapy regimen was initiated on Day 1, and four cycles were administered. Serum tumor markers became negative after two cycles of chemotherapy. (b) After the fourth cycle of the regimen (on Day 116), the patient developed interstitial pneumonia, which improved with corticosteroid therapy. *Bleomycin, etoposide, and cisplatin (BEP) chemotherapy regimen.

**FIGURE 3:**
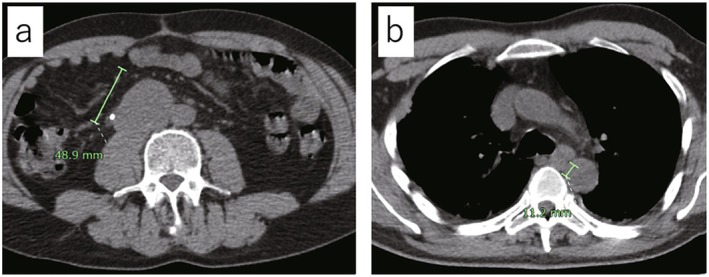
After completion of four cycles of BEP chemotherapy, nodes showed marked regression or complete resolution; however, residual lesions measuring > 1 cm remained in the para‐aortic (a) and mediastinal (b) lymph nodes. The para‐aortic lymph node remained as a residual mass measuring 48.9 mm in maximum diameter.

The patient was diagnosed with a primary retroperitoneal GCT and classified as intermediate prognosis according to the International Germ Cell Cancer Collaborative Group criteria. He had no history of smoking and exhibited normal pulmonary function (%VC, 95.5%; FEV^1^%, 80.8%). The patient received four cycles of bleomycin, etoposide, and cisplatin (BEP) chemotherapy. After two cycles of standard‐dose BEP (bleomycin 30 mg, etoposide 100 mg/m^2^, and cisplatin 20 mg/m^2^), serum tumor markers normalized. Due to febrile neutropenia (FN), cycles 3 and 4 were delayed by 7 days and etoposide was reduced to 80 mg/m^2^. Recurrent FN in all four cycles led to the omission of day‐15 bleomycin (Figure 2). Although metastatic lymph nodes regressed and the left supraclavicular node resolved, residual masses (≥ 1 cm) persisted in para‐aortic and mediastinal nodes (Figure 3). After chemotherapy, the patient developed drug‐induced interstitial pneumonia (IP), which improved with corticosteroid therapy. However, resection of the residual tumor was declined due to the presence of IP.

At 14 months post‐chemotherapy, enlarged retroperitoneal lymph nodes were observed without tumor marker elevation. Deep vein thrombosis (DVT) developed in the area compressed by the tumor, leading to obstruction of the inferior vena cava and initiation of anticoagulant therapy. Histopathological review of the initial biopsy specimen confirmed the presence of a teratoma; therefore, recurrence of teratoma was suspected and curative surgical resection was planned. Retroperitoneal lymph node dissection (RPLND) combined with right nephrectomy was performed. Histopathology of the resected retroperitoneal lesion showed a predominantly sarcomatous component with focal epithelial differentiation, establishing a diagnosis of teratoma with STM. Complete resection with negative margins was achieved.

**FIGURE 4:**
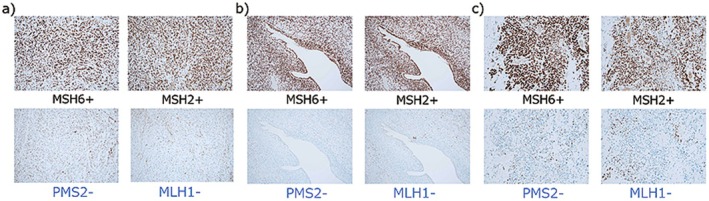
(a) Histopathological section of the resected small bowel showing STM with sarcomatous components. (b) Histopathological section of the resected retroperitoneal mass obtained during RPLND, also showing STM with sarcomatous components. (c) Histopathological section of the retroperitoneal mass obtained by open biopsy at the time of initial diagnosis, revealing a mixed germ cell tumor composed of seminoma, teratoma, and yolk sac tumor. In all sections, IHC showed retained nuclear expression of MSH2 and MSH6, with loss of MLH1 and PMS2 expression—findings consistent with dMMR. Magnification: ×40.

At 6 months, right diaphragmatic crus recurrence invading the iliopsoas muscle and L4 vertebra occurred without tumor marker elevation. Attempted curative resection of the recurrent tumor was abandoned upon discovering intraoperative small bowel invasion, and only the involved intestinal segment was resected. Histopathological examination confirmed that the STM consisted solely of sarcomatous components. MSI testing was subsequently performed on the tissue using the FALCO MSI test kit (FALCO, Kyoto, Japan), which demonstrated an MSI‐high status. Immunohistochemistry (IHC) showed retained nuclear expressions of MSH2 and MSH6, with complete loss of MLH1 and PMS2 expression, findings consistent with mismatch repair deficiency (dMMR). Treatment with pembrolizumab was proposed; however, the patient opted for best supportive care and returned to his home country. Retrospective IHC analyses of tissue specimens obtained at different time points during treatment demonstrated that all histological components identified in the initial biopsy, including seminoma, teratoma, and yolk sac tumor, exhibited dMMR (Figure 4).

## 3 Discussion

This case involved a GCT containing teratomatous primary tumor, followed by the development of STM. STM is a rare but well‐recognized disease state associated with resistance to conventional chemotherapy and poor prognosis [7]. Older patients (≥ 40–50 years) have an increased risk of severe bleomycin‐induced pulmonary toxicity [8]. In the present case, bleomycin‐induced IP resulted in the missed opportunity for residual tumor resection, and prolonged observation without timely surgical resection of residual lesions likely contributed to malignant transformation of the teratomatous component into STM, ultimately resulting in unresectable disease.

Multiple clinical studies of ICI monotherapy have demonstrated limited clinical benefits in GCTs [2, 3, 4, 9, 10, 11]. Therefore, the use of ICI should be restricted to selected cases, particularly those harboring predictive biomarkers such as MSI‐high status. A previous case report described a refractory testicular GCT that exhibited MSI‐high status following fourth‐line chemotherapy, in which pembrolizumab induced a marked clinical response [12]. In this case, MSI‐high was not detected in the primary testicular lesion but was identified in metastatic lung lesions after chemotherapy. Although MSI‐high status was detected in a metastasis with small bowel invasion in our case, retrospective IHC across multiple timepoints revealed dMMR in all components, including the initial biopsy. The reported prevalence of MSI‐high in primary testicular GCT—based on analyses limited to testicular specimens—is approximately 0%–2% [6, 13, 14], which is substantially lower than the 26%–32% observed in studies of refractory GCT, where tissue samples included both testicular and extra‐testicular lesions [13, 15]. Cases in which MSI‐high status is detected only in metastatic lesions may be underrecognized in the real‐world clinical practice. The direct causal relationship between MSI‐high status and pathogenesis of GCT or STM remains unknown. It is possible that genomic instability due to dMMR results in dysregulation of epigenetic genes, resulting in transformation to STM. Further accumulation of data is necessary to assess whether MSI‐high status is associated with carcinogenesis or transformation of GCT to STM.

Given the uncertainty regarding when MSI‐high status may emerge during the disease course, MSI testing in GCT should ideally be performed using tissue obtained from progressive or recurrent lesions, particularly when the clinical course deviates from expectations or when the tumor demonstrates resistance to standard therapy.

## Ethics Statement

Ethical approval was not required for this case report in accordance with institutional policies.

## Consent

Informed consent was obtained from the patient for publication of this case report and accompanying images.

## Conflicts of Interest

The authors declare that they have no conflicts of interest relevant to this article. Dr. Shohei Ishida is an Editorial Board member of International Journal of Urology and a co‐author of this article. To minimize bias, he was excluded from all editorial decision‐making related to the acceptance of this article for publication.

## Data Availability

The data that support the findings of this study are available from the corresponding author upon reasonable request.

## References

[1] T. Nakamura , T. Ueda , M. Oishi , et al., “Importance of Continuous Sequential Chemotherapy and Multimodal Treatment for Advanced Testicular Cancer: A High‐Volume Japanese Center Experience,” Medicine (Baltimore) 94 (2015): e653, 10.1097/MD.0000000000000653. 25789960 PMC4602480

[2] N. Adra , L. H. Einhorn , S. K. Althouse , et al., “Phase II Trial of Pembrolizumab in Patients With Platinum Refractory Germ‐Cell Tumors: A Hoosier Cancer Research Network Study GU14‐206,” Annals of Oncology 29 (2018): 209–214, 10.1093/annonc/mdx680. 29045540

[3] A. M. Tsimberidou , H. H. Vo , V. Subbiah , et al., “Pembrolizumab in Patients With Advanced Metastatic Germ Cell Tumors,” Oncologist 26 (2021): 558–e1098, 10.1002/onco.13682. 33491277 PMC8265349

[4] S. Zschäbitz , F. Lasitschka , B. Hadaschik , et al., “Response to Anti‐Programmed Cell Death Protein‐1 Antibodies in Men Treated for Platinum Refractory Germ Cell Cancer Relapsed After High‐Dose Chemotherapy and Stem Cell Transplantation,” European Journal of Cancer 76 (2017): 1–7, 10.1016/j.ejca.2017.01.033. 28262583

[5] M. Yarchoan , A. Hopkins , and E. M. Jaffee , “Tumor Mutational Burden and Response Rate to PD‐1 Inhibition,” New England Journal of Medicine 377 (2017): 2500–2501, 10.1056/NEJMc1713444. 29262275 PMC6549688

[6] F. M. Cárcano , A. H. Lengert , D. O. Vidal , et al., “Absence of Microsatellite Instability and BRAF (V600E) Mutation in Testicular Germ Cell Tumors,” Andrology 4 (2016): 866–872, 10.1111/andr.12200. 27153176

[7] P. Giannatempo , G. R. Pond , G. Sonpavde , et al., “Treatment and Clinical Outcomes of Patients With Teratoma With Somatic‐Type Malignant Transformation: An International Collaboration,” Journal of Urology 196, no. 1 (2016): 95–100, 10.1016/j.juro.2015.12.082. 26748165

[8] A. B. Simpson , J. Paul , J. Graham , and S. B. Kaye , “Fatal Bleomycin Pulmonary Toxicity in the West of Scotland 1991–95: A Review of Patients With Germ Cell Tumours,” British Journal of Cancer 78 (1998): 1061–1066, 10.1038/bjc.1998.628. 9792151 PMC2063168

[9] M. Mego , D. Svetlovska , M. Chovanec , et al., “Phase II Study of Avelumab in Multiple Relapsed/Refractory Germ Cell Cancer,” Investigational New Drugs 37 (2019): 748–754, 10.1007/s10637-019-00805-4. 31152292

[10] A. Necchi , P. Giannatempo , D. Raggi , et al., “An Open‐Label Randomized Phase II Study of Durvalumab Alone or in Combination With Tremelimumab in Patients With Advanced Germ Cell Tumors (APACHE): Results From the First Planned Interim Analysis,” European Urology 75 (2019): 201–203, 10.1016/j.eururo.2018.09.010. 30243800

[11] T. Kawahara , K. Kawai , T. Kojima , et al., “Phase II Trial of Nivolumab Monotherapy and Biomarker Screening in Patients With Chemo‐Refractory Germ Cell Tumors,” International Journal of Urology 29 (2022): 741–747, 10.1111/iju.14885. 35462438 PMC9545636

[12] K. Kawai , A. Tawada , M. Onozawa , et al., “Rapid Response to Pembrolizumab in a Chemo‐Refractory Testicular Germ Cell Cancer With Microsatellite Instability‐High,” Oncotargets and Therapy 14 (2021): 4853–4858, 10.2147/OTT.S323898. 34584425 PMC8464369

[13] F. Honecker , H. Wermann , F. Mayer , et al., “Microsatellite Instability, Mismatch Repair Deficiency, and BRAF Mutation in Treatment‐Resistant Germ Cell Tumors,” Journal of Clinical Oncology 27 (2009): 2129–2136, 10.1200/JCO.2008.18.8623. 19289622

[14] A. Necchi , G. Bratslavsky , R. J. Corona , et al., “Genomic Characterization of Testicular Germ Cell Tumors Relapsing After Chemotherapy,” European Urology Focus 6 (2020): 122–130, 10.1016/j.euf.2018.07.013. 30025711

[15] F. Mayer , H. Wermann , P. Albers , et al., “Histopathological and Molecular Features of Late Relapses in Non‐Seminomas,” BJU International 107 (2011): 936–943, 10.1111/j.1464-410X.2010.09631.x. 20955261

